# Biomonitoring of complex occupational exposures to carcinogens: The case of sewage workers in Paris

**DOI:** 10.1186/1471-2407-8-67

**Published:** 2008-03-06

**Authors:** Hamzeh Al Zabadi, Luc Ferrari, Anne-Marie Laurent, Aziz Tiberguent, Christophe Paris, Denis Zmirou-Navier

**Affiliations:** 1 INSERM-ERI 11, Nancy University Medical School, 9 av de la Forêt de Haye, BP 184, 54505 Vandoeuvre-les-Nancy Cedex, France; 2 Faculty of Pharmacy of Nancy, 5 rue Albert Lebrun, 54000 Nancy, France; 3 Hygiene Laboratory of the City of Paris, 11, rue George Eastman, 75013 Paris, France; 4 Department of Occupational Medicine, Municipality of Paris, 44 rue Charles Moureu, 75013 Paris, France

## Abstract

**Background:**

Sewage workers provide an essential service in the protection of public and environmental health. However, they are exposed to varied mixtures of chemicals; some are known or suspected to be genotoxics or carcinogens. Thus, trying to relate adverse outcomes to single toxicant is inappropriate. We aim to investigate if sewage workers are at increased carcinogenic risk as evaluated by biomarkers of exposure and early biological effects.

**Methods/design:**

This cross sectional study will compare exposed sewage workers to non-exposed office workers. Both are voluntaries from Paris municipality, males, aged (20–60) years, non-smokers since at least six months, with no history of chronic or recent illness, and have similar socioeconomic status. After at least 3 days of consecutive work, blood sample and a 24-hour urine will be collected. A caffeine test will be performed, by administering coffee and collecting urines three hours after. Subjects will fill in self-administered questionnaires; one covering the professional and lifestyle habits while the a second one is alimentary. The blood sample will be used to assess DNA adducts in peripheral lymphocytes. The 24-hour urine to assess urinary 8-oxo-7, 8-dihydro-2'-deoxy-Guanosine (8-oxo-dG), and the in vitro genotoxicity tests (comet and micronucleus) using HeLa S3 or HepG2 cells. In parallel, occupational air sampling will be conducted for some Polycyclic Aromatic Hydrocarbons and Volatile Organic Compounds. A weekly sampling chronology at the offices of occupational medicine in Paris city during the regular medical visits will be followed. This protocol has been accepted by the French Est III Ethical Comitee with the number 2007-A00685-48.

**Discussion:**

Biomarkers of exposure and of early biological effects may help overcome the limitations of environmental exposure assessment in very complex occupational or environmental settings.

## Background

Sewage workers provide an essential service in the protection of public and environmental health. In large cities, sewage is composed of organic residues but also incorporate a wide variety of chemicals produced by roadways scrubbing by rain, water from office and industrial facilities, domestic activities (remainders of painting, drugs, pesticides used indoor, etc). As a result of their contact with wastes, sewage workers are exposed to complex mixtures of toxicants including pathogens, heavy metals, chlorinated organic solvents like chloroform, dichloroethane, perchloroethanol, other solvents (benzene, toluene), aldehydes, nitrosamines, pesticides, dyes, polychlorobiphenyls, and polycyclic aromatic hydrocarbons (PAH) [1-3]. Many of these compounds are known or suspected to be genotoxics and/or carcinogens [4-6], which suggests that those workers may be subject to elevated risk of cancer.

Previous studies have indicated an increase in the incidence of cancer among sewage workers [7-10]. Analyses on specific cancer sites have reported excess numbers of laryngeal, primary liver cancer [8,10], cancer of the prostate gland, nose and nasal sinuses cancers, stomach [9], central nervous system [11], and bladder cancers [12]. However, these data exhibit conflicting results [8,9]. A more recent mortality study among the sewage workers of Paris published in 2006 assessed their cause-specific mortality from 1970 until 1999 [7]. A slight but significant excess in mortality was found (SMR = 1.25, 95% CI; 1.15–1.36) in particular from cancer mortality (SMR = 1.37, 95% CI; 1.20–1.56), with a suggested excess for oesophagus, liver, pleura and the brain cancers albeit not significant. However, this study didn't measure personal or workplace exposures; it used only qualitative information gathered by a questionnaire and the computerized register of the employees.

As exposure of sewage workers implies contact with multiple potent genotoxics at varying levels (by concentration, time and location) and routes of exposure (by inhalation, dermal and ingestion) [9], characterizing and quantifying it are extremely difficult, and trying to relate adverse outcomes to single toxicant is inappropriate. However, usage of biomarkers to study the association between exposure and early biological genotoxics effects seems more relevant in this setting [5]. These findings may explain that previous studies among sewage workers exhibited conflicting results; some were biased by many confounding factors; others relied on qualitative and/or a questionnaire data; while others used urine or blood samples to evaluate the exposure without workplace measurements. However, sewage workers might be exposed to many agents that may interact with one another resulting in an immeasurable amount of different chemicals. Rather than trying to describe this immense array of exposures or pursuing the goal to relate the biological health outcomes to specific compound, it might be more reasonable to look for unspecific early effects. Further, changes in the composition of the sewage system over-time may affect the level and character of worker's exposure longitudinally. Thus, assessment of genotoxics at only one point in time may not represent long term occurrence of these substances in the body [13]. However, workplace environment sampling at various locations over-time when the biological specimens were taken might be more representable and would further support the link between occupational sewage exposure and the appearance of genotoxics in both sample types (urine and blood).

In order to precise/assess such exposures to genotoxics/carcinogens compounds, urine genotoxicity has been widely used as a noninvasive method to evaluate recent exposure among populations exposed to environmental and/or workplace-related complex mixtures of chemicals [14-16]. *In Vitro *comet [14] and micronucleus [17] assays are among the most widely-used biomarkers of urine genotoxicity for monitoring the risk of DNA damage that stems from occupational and environmental exposures to genotoxics. Comet assay is a sensitive technique, can detect DNA damage in terms of double and single-strand breaks, and alkaline-labile sites [18,19]. Micronucleus test is a reliable biomarker of irregularity of genetic material due to non-specific genotoxic exposure [20,21].

Of the complex mixtures to which sewage workers are exposed, are PAHs and other genotoxic chemicals that are metabolized by and induce the expression of cytochrome P450 enzymes (e.g. CYP1A2) [22-24]. The CYP1A2 enzyme is involved in the metabolic activation of a wide range of chemicals and carcinogens like PAHs and aromatic amines [23,24]. Its activity has been shown to be increased by smoking, ingestion of charbroiled meat, cruciferous vegetables, PAHs and PCBs exposures [23,25-28]. The catalyzed metabolism by CYP1A2 can generate ROS which might lead to oxidative DNA damage [22,29,30]. This damage has been associated with an increased risk of cancer generally ascribed to DNA adducts [22,31]. Thus, measurement of CYP1A2 activity in vivo may be an important tool to assess the exposure to chemical carcinogens and cancer risk. PAHs related DNA-adducts measured by ^32^P- postlabeling technique is frequently described as the biomarker of choice [32-34]. Oxidative DNA damage may be also important in carcinogenesis since the DNA base lesions, such as 8-oxodG, are abundant and highly mutagenic [35,36]. However, DNA repair via nucleotide and base excision processes leads to elimination and excretion of 8-oxodG in urine quantitatively without metabolism [37-40]. Urinary excretion of 8-oxodG is the most widely used noninvasive urinary biomarker of oxidative stress and its measurement in urine has been proposed to assess whole-body oxidative DNA damage [41,42].

The CYP1A2 was shown to be responsible for the 3-demethylation of caffeine, which is the initial major step in the biotransformation of caffeine in human's body [43]. Urinary metabolites of dietary caffeine is the most noninvasively-used method in the assessment of CYP1A2 activity [44-46]. Recent studies have demonstrated that the polymorphism of CYP1A2 could be critical in investigating the induction of the enzyme [47]. The -163C>A (allele CYP1A2*F) polymorphism has been associated with higher enzyme inducibility by smoking [48]. Even if the clinical relevance of this polymorphism remains controversial [47], it is necessary to assess it for a good interpretation of the caffeine metabolism data.

This project is interested in an association of simple, early and non-invasive biomarkers intended to highlight exposure to cocktails of undefined toxic substances having genotoxic properties. We propose to carry out a cross sectional study comparing a particularly exposed category of workers to multiple professional pollutants (Parisians sewage workers) with a non-exposed professional category workers (municipality office workers) by using biomarkers of exposure and early biological effects. The biomarkers dedicated for this study are; the comet and micronucleus tests, which seek the presence of genotoxics in the urine. A second group of biomarkers highlights early effects of these substances; the caffeine test, relevant in the event of exposure to PAHs [49], DNA-adducts in the lymphocytes, a biomarker of early effect indicating the exposure to gentoxics, and urinary 8-oxo-dG, a biomarker of early effect, corresponding to DNA oxidative stress [50]. Our primary objective is to study if the exposed present an increased risk of genotoxic lesions, compared to the nonexposed. The secondary objective is to evaluate the early effects of an exposure to complex genotoxic agents. To achieve these objectives we will; (1) analyze the urine for DNA damage and genotoxicity (using in vitro comet and micronucleus assays and analysis of oxidative stress through 24 h urinary 8-oxodG), (2) analyze peripheral blood lymphocytes for DNA-adducts by ^32^P-post labelling technique, (3) assess personal exposure to PAHs and VOCs in the workplace environment, and (4) evaluate the PAHs exposure through assessment of CYP1A2 activity by urinary metabolites of dietary caffeine.

**Figure 1:**
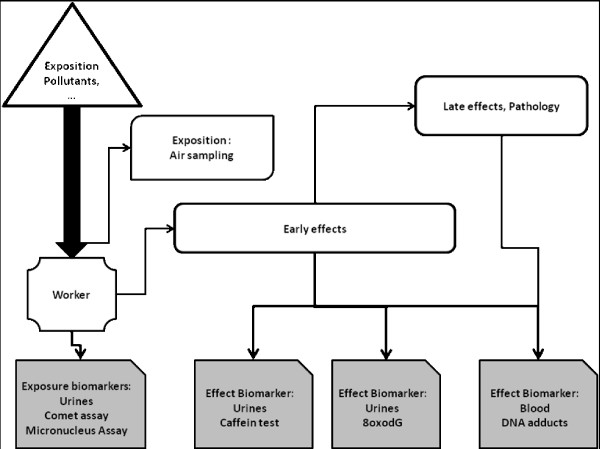
Schematic diagram of progression from exposure to disease and the study assessment tools and biomarkers.

The study hypothesis is that exposure of the sewage workers to multiple genotoxics leads to an increase in certain biomarkers of exposure and other biomarkers of early biological effects. The validation of our hypothesis through these biomarkers, would allow the estimation of the total personal exposure to complex mixture of toxic chemicals from different exposure pathways (lungs, skin, and GIT), and different sources (air, diet, lifestyle or occupation), whereas the traditional epidemiological studies don't. Figure 1 presents the theoretical-overview of development from exposure to disease and the study assessment biomarkers.

## Methods/Design

### Study design, population and setting

This cross sectional study will compare an exposed population (under-ground sewage workers) to a control group (office workers). Both groups are from Paris municipality workers and selected among occupational categories with similar socio-economic status. Participation will be voluntary. Subjects will be current nonsmokers since at least 6 months, aged (20–60) years old, being employed during at least the same period, have no history of chronic or recent illness (diabetes, influenza for example) and are not taking any medication (omeprazole for instance) that could interfere with the study results. As sewage workers are mostly males, the study population will be only of males. The study will be conducted in the framework of regular occupational medical visits. All interviews and primary procedures will be taken place at the offices of occupational medicine in Paris city.

### Ethical consideration

The study protocol was approved by the local ethical committees (CPP, N°2007-A00685-48). All participants will be given an explanation of the nature of the study, and a signed informed consent will be obtained.

### Sampling chronology

Table 1 shows the weekly sampling chronology of the study participants. Briefly, controls will be frequency-matched for age with sewage workers with a 1 to 1 ratio. After at least three consecutive days of work a 24 h urinary sample will be collected from all participants (starting at 9:00 a.m). Subjects will receive a urine collecting bottle and written/oral information describing urine collection. After given their urine samples at 9:00 a.m of the next day (Friday), the exposed will undergo medical examination by occupational health physicians. Blood samples will then be taken by nurses. Thereafter, they will receive a cup of decaffeinated coffee added with 110 mg of caffeine. Three hours later, a urine sample will be taken, from which three aliquots (200 μl each) will be collected to assess the urinary caffeine metabolites and the corresponding CYP1A2 activity. During these 3 hours, subjects will fill in two self-administered questionnaires under the supervision of study researchers. A professional one covering socio-demographic factors, non-occupationally exposures (especially PAHs-related: commuting means, area of residence and indoor sources), medical history, lifestyle (smoking history including passive smoking exposure, alcohol and medications) and other confounders. The other is an alimentary questionnaire collecting detailed-data on diet habits [51]. For the control group, the sequence will be the same but start at 13:30 p.m.

**Table 1:** Morning sampling chronology of study participants.

*9h00	**9h30**	**10h00-12h00**	**12h30**	**13h00**	**13h30**
24h urine collection					
Pre-treatment of 24h urine samples					
	Cup of Coffee				
Medical visit					
Blood sample					
	Isolation and pre-treatment of lymphocytes				
	Biological tests (urea, creatinine)				
	Questionnaires				
			3h urine collection		
				Pre-treatment of 3h urine samples	
			Thanks	Departure	

*Friday of each week. In each study week, 16 participants will be sampled (8 exposed in the morning and 8 non-exposed in the corresponding afternoon).

Blood and urine samples will be processed on the same day as described further. For the 48 h before and during the 3 hours of the caffeine test, subjects will be asked to avoid diets or cooking procedures known to increase CYP1A2 activity or elicit urinary mutagenicity (e.g., cruciferous vegetables; charcoal-broiled or grilled meat) or inhibit CYP1A2 (e.g., grapefruit). They will also be asked to refrain from consuming alcoholic drinks and beverages containing methylxanthines and to avoid massive physical activity as it could increase DNA damage [52,53]. In table 1 we present the morning sampling chronology for the exposed participants.

### Occupational atmospheric sampling

During the first part of the week, before coming to the medical examination, the air of the working places will be collected to be assessed for their COV or HAP content.

### Sample size

Sample size was calculated for a type ^2 ^error (α) of 5% and power expectation of 80%. In a non-exposed population, urines are not mutagenic in theory, and both genotoxicity tests should be negative. Thus, if the expected prevalence in the control is 1%, a number of 75 subjects in each group are sufficient to highlight a prevalence of the anomalies of 17% in the exposed. The number of subjects necessary for DNA-adducts study is similar. For urinary 8-oxo-dG, the expected value in reference (control) population is nearly 10.78 ± 6.6 (Mean ± SD) nmole/24h, [54]. Thus 75 subjects in each group are sufficient to detect 18% modification of this value. For CYP1A2 activity, the urinary "molar concentration ratio of 1, 7-dimethylurate plus paraxanthine over caffeine" measured in a reference population is 5.6 ± 1.5 (Mean ± SD) [55], so 75 subjects in each group allow to detect a modification of 13% of this ratio.

As describe above, 16 subjects (8 exposed and 8 non-exposed) will be sampled Each week,. This will result in 10 weeks of sampling procedures to complete data collection according to study sample size of 75 subjects in each group. Each corresponding afternoon, 8 non-exposed participants will follow a similar sampling procedure.

The Parisians sewage workers are nearly 400 individuals, recruitment will be comfortable, even after exclusion of smokers (approximately 45%) [7].

### Experimental protocol and technique

#### Isolation of lymphocytes

This will be carried out using Ficoll gradient centrifugation method of Bøyum [56] with few modifications according to the study conditions. Briefly, 25 ml of freshly obtained venous blood will be collected on anticoagulant (EDTA) and diluted with an equal volume of standard balanced salt solution and layered carefully over Ficoll-Paque Plus density gradient medium, without intermixing, in a centrifuge tube. After centrifuging at room temperature (400 g for 30–40 min), drawing of the upper layer by a clean Pasteur pipette will be done leaving the lymphocytes layer undisturbed at the interface. The upper layer which contains the plasma will be saved for later usual clinical chemistry tests. Using a clean Pasteur pipette the lymphocytes layer will be harvested from the interface and transferred to a clean centrifuge tube. Then it will be centrifuged twice (60–100 g for 10 min at 18–20°C) in a balanced salt solution to wash the lymphocytes and remove any remnants of platelets. The lymphocytes will be suspended with 10% DMSO, coded, and frozen at -80°C until extraction of DNA.

#### Extraction of DNA

Frozen lymphocytes suspensions will be thawed in a 37°C water bath with gentle agitation. DNA extraction will be carried out using a standard phenol-chloroform method including treatment with RNAses as described elsewhere [57]. DNA purity will be checked by determination of UV spectra between 228 and 300 nm (associated with ratio values: 1.8<A260/A280<1.95 and A260/A230>2.3) and the DNA concentration will be deduced from the A260, as described [58]. DNA solutions will be divided into three portions and frozen at -80°Cin glass vials.

#### Polymerase chain reaction "PCR" analysis of the CYP1A2

The polymorphism of CYP1A2 will be assessed by real-time polymerase chain reaction and melting curve analysis, as described by Casley and LeBlanc-Westwood [59]. Reactions will be carried out in 20 μL volumes containing 3.5 mM MgCl_2 _and 50 pg genomic DNA, using Fast Start DNA master mix for hybridization probes from Roche Diagnostics. All conditions will be adapted from Casley and LeBlanc-Westwood [59].

#### Analysis of DNA-adducts

DNA-adducts will be analyzed by ^32^P-postlabelling assay as described [60,61], using Nuclease P1 for enrichment, with modifications from le Goff [57]. Briefly, 5 μg of DNA will be digested, then μCi γ-^32^P-ATP. Separation will be achieved on thin layer chromatography. Autoradiograms will be obtained after exposure of Kodak Biomax film to the TLC-plates. Each sample will be analyzed two times and in at least two different experiments. The detection limit will be fixed at 0.02 × 10^-10^, i.e. half of the lowest quantifiable Relative Adduct Level (RAL) value. For qualitative analysis, the mean number of adducts per individual will be calculated.

#### Pre-treatment of urine samples

The volume of the 24h urine collected in sterile plastic urine collection bottles will be measured immediately and expressed per subject and body weight. Then, three 10 ml aliquots will be coded and frozen at -20°C for 8-oxodG analysis. Another up to 100 ml aliquots will be coded and frozen at -20°C for organic extraction and genotoxicity tests. Both samples will be transferred to the laboratory of analysis (within the same day). The concentration of 8-oxodG in urine stored at -20°C was shown to be constant for at least 3 years [62].

### Measurement of 8-oxodG concentration in 24h urine

This will be done as described [63]. Briefly, frozen urine samples will be thawed at 37°C for 25 min, mixed and cooled to room temperature. HPLC separation will be performed on a C18 HPLC column (150 × 2 mm, 5 μ) protected by a C18 guard column (10 × 2 mm, 5 μ). The mobile phase for urine samples will be 10 mM ammonium formate, adjusted to pH 3.75 with formic acid and 2% acetonitrile. Electrospray will be performed in the positive ion mode. A stable isotopically marked internal standard of 8-oxodG will be used ([^15^N_5_] 8-oxo-dG) (for details, see reference 63).

### Urine organic extracts

This will be carried on Sep-Pak C18 cartridges (Waters Associates, Inc) adsorption chromatography as described [64] with some modifications. Briefly, frozen urine samples will be thawed at room temperature and filtered through Whatman filter paper No. 1. Then it will be adjusted to pH 7 using 0.1 M NaOH. The cartridge will firstly be washed 3 times with 3 ml of absolute methanol and 3 ml of ultra-pure water successively before preparation of the columns. Then, the cartridge will be loaded with urine using a glass powder funnel on the column to facilitate the loading process. All operations will be at room temperature. The column will then be washed 3 times with 10 ml distilled water in order to eliminate the residual urine and histidine. The adsorbed components will then be eluted with methanol (5 ml/100 ml urine) into glass test tube. The eluate will be dried at 40°C under a nitrogen stream until complete dryness. Then, the residue will be dissolved in DMSO (0.4 ml/100 ml urine) and stored at -20°C until analysis of genotoxicity tests.

### Cell culture

For the two tests (comet and micronucleus) two cell lines will be used. HeLa S3 cellular line cells will be used (ECACC, catalog number 87110901, adherent cells of human cervical carcinoma). Hep G2 is a perpetual adherent cell line which was derived from the liver tissue of a 15 year old caucasian male with a well differentiated hepatocellular carcinoma (ATCC, catalog number HB-8065).

### Comet assay "Single Cell Gel Electrophoresis" (SCGE)

The urine extracts kept at -20°C will be thawed and warmed to room temperature shortly before the assay. Comet assay will be performed basically according to Sing et al. 1988 [65], with modifications according to Muller et al. 2000 [66]. Briefly, the cells will be incubated with the organic extract of urine (200 μl) during 24h (typical division duration of these cells). Viability of cells will be determined by trypan blue test. Microscopic slides will be precoated with 100 μl of agarose (1%). The slides will be gently immersed in ice-cold freshly lysis solution and will be covered with fresh electrophoresis buffer for 20 min and placed in a horizontal electrophoresis unit tank filled with new fresh electrophoresis buffer. After electrophoresis, they will be washed with a freshly made neutralizing buffer and stained with 50 μl ethidium bromide solution. They will then be examined for analysis of DNA migration under a fluorescence microscope (Olympus BX-40, Olympus, Japan) using a computerized image analysis system (Komet 5, Kinetic Imaging). Two slides will be analyzed for each sample with fifty cells scored in each slide. Olive tail moment will be used for analysis of results [67].

### Micronucleus assay

It will be performed according to the standard protocol of the International Workshops on Genotoxicity Test Procedures [17,68,69]. Briefly, after the initial screening; well-prepared slides will be scored using a high power magnification (400–1000 folds) with both bright field and phase-contrast microscope. Frequency of micronucleated cells will be evaluated by the number of cells containing one or more micronuclei (but less than 5). The induction factor will be calculated by dividing treated values by the control ones. Chi-square will be used for the comparisons and when P value is < 0.05 the concentration will be considered positive.

### Assessment of CYP1A2 activity by urinary caffeine metabolites

Subjects will be instructed to empty their bladder. Then they will receive a cup of decaffeinated coffee added with 110 mg caffeine. Three hours later, a urine sample will be collected and transferred to tube with 1 ml HCl, pH 3.5. Samples will be coded and frozen at -20°C and then transferred to the laboratory of analysis (within the same day), where it will be stored at -20°C until HPLC analysis. Caffeine and its metabolites will be extracted as described [55]. Briefly, the concentrated residue will be dissolved in 800 μl of 0.05% acetic acid and filtered through a 0.45-μm filter. Here, 100 μl of the filtrate will be injected into HPLC column. Caffeine and its metabolites will be analyzed using an HPLC system as described elsewhere [70]. The metabolites will be identified and quantified by UV detector with a computerized photodiode array detector as compared with definite standards [1,7-dimethylurate (17 U), 1,7-dimethylxantine (17X), and 1,3,7-trimethylxanthine (137X)]. To assess CYP1A2 activity, urinary molar concentration ratio index [17U+ 17X/137X] will be used as it reflects caffeine 3-demethylation activity in this phenotyping procedure [71,72].

### Occupational air sampling

The targeted indicators will be VOCs and PAHs. They have been selected because they are present in the confined environments of the sewers while also emitted by automobile traffic, hence present in ambient and indoor atmospheres, and because they are of health significance.

Since the sewage system is deprived of electricity, air sampling will be carried out using battery-powered devices or passive samplers. The sampling procedure will strive at evaluating exposure near the breathing zone. However, not to disturb the sewage workers, measurements will be done by a companion worker (or a study personnel) who will accompany each studied team and carry the sampling equipment in a back bag. For the reference population (office workers) the same type of sampling materials will be placed in a bag located in the working area, for example on a desk.

### Measurement of Volatile Organic Compounds (VOCs)

Collection of VOCs will be carried out on thermal desorption sorbent tubes exposed during the sewage workers worktime from Monday to Thursday and the sampler is recapped after every exposure. Analysis will be carried out by coupling gas chromatography and mass spectrometry. As work in sewage system takes place in a wet environment, a sorbent tube not very sensitive to moisture will be chosen. The list of the selected indicators will be at least the substances measured inside residences within the framework of the national inventory carried out by the Observatory of Indoor Air Quality (OIAQ) in more than 560 French residences. These data were published in November 2006 [73] and can be used as reference values. It is probable that most of compounds found in the residences are also present in the air of the offices. The basic list is as follows:

- Alkanes: decane, undecane

- Monocyclic aromatic hydrocarbons: benzene, toluene, meta and para-xylenes, orthoxylene, 1, 2, 4-trimethylbenzene, styrene

- Chlorinated hydrocarbons: trichloroethylene, tetrachloroethylene, 1–4-dichlorobenzene.

The tubes used for sampling will be analyzed at the Paris city hygiene laboratory on a chain including a thermal desorption module on line with a chromatograph in gas phase equipped with a capillary column and coupled to a mass spectrometer (Quadripole). Quantitative analysis will be carried out on the basis of ion extracts and a range of calibration prepared by doping a lot of sorbent tubes with various quantities of a mixture of the selected VOCs. The first samples (2 to 3) will be devoted to the qualitative analysis (screening) of the chromatographic profiles with the aim to adjust the list of targeted compounds.

### Measurement of Polycyclic Aromatic Hydrocarbons

They will be collected with personal air samplers allowing the simultaneously trapping of the volatile and the particulate phases (in case of heavy loss of charge due to high charged XAD2 resin, it will be necessary to carry out two distinct samples, one collecting only gaseous PAHs, the second one for particulate PAHs). The head of the samplers will consist of a cassette containing a filter, to collect particles coupled to a marketed tube filled with XAD2 resin or polyurethane foam. Air will be drawn using a constant flow sampling pump at a calibrated flow-rate of 2 L/min.

Duration of sampling will be at least equal to the daily worktime. However, four consecutive days of cumulative sampling may be necessary because concentrations are expected to be low. In this case, filter will be preserved in an aluminum sheet to avoid photochemical transformations. A first series of measurements will allow determination of the minimal duration of exposure for an acceptable quantification limit.

Thirteen PAHs will be measured: phenanthrene, anthracene, fluoranthene, pyrene, benzo(a)anthracene, chrysene, benzo(j)fluoranthene, benzo(b)fluoranthene, benzo(k)fluoranthene, benzo(a)pyrene, dibenzo(a,h)anthracene, benzo(g,h,i) perylene, indeno-pyrene. PAHs will be extracted at the laboratory by a solvent in a pressurized cell, so the extracts will be concentrated in an automatic evaporator. They will be analyzed by high HPLC associated with fluorimetric detection. A binary elution gradient consisting of water and acetonitrile will be used to separate the different PAHs. Several couples of wavelengths of excitation and emission will be selected to optimize the sensitivity of the response of the compounds and to limit the chromatographic interferences. The quantitative analysis will be carried out according to the response of standard solutions that are prepared from a marketed mixture of the selected PAHs.

### Statistical analysis

For each parameter, data will be compared between the exposed and non-exposed groups. Data will be tested for homogeneity of variance and normality after variable transformation if appropriate. Two-tailed Student's t-test will be used for group and/or sample comparisons relative to DNA-adducts level. Fisher's exact test will also be used for comparison of DNA-adducts pattern distributions between groups. Linear regression analysis will be used for quantitative variables, adjusting for parameters that reflect exposures in the questionnaires. For other parameters, ANOVA will be performed. Correlation analysis of DNA-adducts levels and urine genotoxicity with qualitative parameters will be evaluated by Spearman tests. Potential confounding factors, like age, socioeconomic or passive smoking will be studied, mainly by evaluating their distribution in both groups and by looking for possible association with DNA-adducts levels, urine genotoxicity or caffeine metabolism tests. The influence of confounding factors will be determined by multiple logistic regression after a check of normality (Kolmogorov Smirnov's test). The analysis will be processed by the statistical software SAS (SAS Inc., Version 8.02).

## Discussion

This study aims to investigate the carcinogenic risk associated with occupational exposure of sewage workers to complex chemical mixtures. While the comet assay can detect DNA reparable lesions or alkali-labile sites, micronucleus can detect fixed mutations that persist at least one mitotic cycle [74]. Positive results in the comet don't necessarily correspond to positive results in the micronucleus, especially when genotoxic exposure is small. Thus, the combination of both assays might be more accurate and reasonable. Urinary excretion of 8-oxodG is a repair product of oxidative DNA damage and under the usual steady conditions it reflects the general average risk of a promutagenic oxidative stress in DNA of all tissues and organs [75]. Further, DNA-adducts in peripheral lymphocytes is considered as a good biomarker when studying the early effect of genotoxic exposures in humans [76,77].

This study is limited by its cross sectional design where systematic differences between exposed and non-exposed could cause under or overestimation of the risk, as exposed subjects may be more motivated to participate than non-exposed. However, genotoxicity tests (urine and lymphocytes) are not likely to be affected by the subjects' interest to the study. Moreover, choosing office workers as a control group may alleviate sources of strong bias such as "healthy worker effect" and social class differences, as both groups belong to the same socioeconomic class. Further, urine genotoxicity is a short-term measure that reflects exposure 24 to 72h before collection [78] and our blood samples will be taken at rest. Airborne assessment will assess exposure by inhalation only, thus possibly misclassifying exposure both quantitatively and qualitatively. Using biomarkers of exposure and of early effects aims to overcome this shortcoming in view to assess the risk. Some difficulties might stem fromthe tiny amounts of promutagens in urine and the presence of urinary histidine, that leads to false positive results. Filtration and concentration of urine might help to solve these problems [64]. Finally, day to day variability in laboratory procedures will be calculated and estimated by analysis.

To summarize, sewage workers are exposed to multiple chemicals from multiple pathways resulting in a complicated matrix of exposure to chemicals and concentrations. In this complex chemical exposure setting, this study combines biological sampling, both in blood and urine, to assess biomarkers of exposure and of early biological effects. These biological indicators will be scaled with results of workplace environment air sampling that will be conducted in parallel. Such biomarkers of exposure and of early biological effects may help overcome the severe limitations of environmental exposure assessment in very complex occupational or environmental settings. If shown discriminating in the framework of this study population, these non-specific biomarkers might be used to assess the genotoxic risk in other populations also experiencing complex exposures.

## Abbreviations

CI–Confidence interval, CYP1A2–Cytochrome P1A2, DNA–Deoxyribo-nucleic Acid, EDTA–Ethylene-diamine-tetra-acetic acid, HCl–Hydrochloric acid, HPLC–High performance liquid chromatography, MgCL2–Magnesium chloride, MN–Micronucleus, PAHs–Polycyclic aromatic hydrocarbons, SMR–Standardized mortality rate, VOCs–Volatile organic compounds, 8-oxodG–8-Oxo-7, 8-Dihydro-2^'^-deoxy-Guanosine

## Competing interests

The author(s) declare that they have no competing interests.

## Authors' contributions

HA drafted the manuscript. LF, DZN and CP participated in the design and coordination of the study protocol and helped to draft the manuscript. Other authors reviewed the manuscript, provided further contributions and suggestions. All authors read and approved the final manuscript.

## Pre-publication history

The pre-publication history for this paper can be accessed here:


